# Diabetes mellitus among HIV-infected individuals in follow-up care at University of Gondar Hospital, Northwest Ethiopia

**DOI:** 10.1136/bmjopen-2016-011175

**Published:** 2016-08-18

**Authors:** Solomon Mekonnen Abebe, Assefa Getachew, Solomon Fasika, Mulugeta Bayisa, Abayneh Girma Demisse, Nebiyu Mesfin

**Affiliations:** 1 Institute of Public Health, College of Medicine and Health Science, University of Gondar, Gondar, Ethiopia; 2 School of Medicine, College of Medicine and Health Science, University of Gondar, Gondar, Ethiopia

## Abstract

**Objective:**

To assess the prevalence of diabetes mellitus (DM) and associated factors among HIV-infected adults in northwest Ethiopia.

**Design:**

Hospital-based cross-sectional study.

**Setting:**

HIV clinic of the University of Gondar Hospital, Ethiopia.

**Participants:**

All HIV-infected adults who visited the HIV clinic from December 2013 to the end of February 2014 were the source population.

**Measures:**

A structured and pretested questionnaire incorporating the WHO STEPwise approach was used. A multivariate logistic regression analysis was applied to assess factors associated with DM.

**Results:**

The overall prevalence of type 2 DM was 8% (95% CI 5.5% to 10.5%). The prevalence of DM was higher (13.2%; 95% CI 8.0% to 18.3%) among subjects receiving pre-antiretroviral treatment (pre-ART) than among those taking ART (5.1%; 95% CI 2.6% to 7.6%). Thirteen (35.1%) of the DM cases were newly identified during the study. Obesity (adjusted OR (AOR) 6.55; 1.20 to 35.8), hypertension (AOR 3.45; 1.50 to 7.90), being in the pre-ART group (AOR 4.47; 1.80 to 11.08), hypertriglyceridaemia (AOR 2.24; 1.02 to 49.5) and tertiary-level education (AOR 11.8; 2.28 to 61.4) were associated with DM.

**Conclusions:**

Overall DM prevalence was high, particularly among subjects in the pre-ART group. More educated, hypertensive and obese HIV-infected adults were more likely to have DM as a comorbidity. Health policy and the clinical management of HIV-infected individuals should take into account the rising DM.

**Strengths and limitations of this study**

- Our study assessed the prevalence of diabetes mellitus (DM) and factors associated with it. DM is known to be one of the most common non-communicable diseases in patients living with HIV.
- Our findings provide important information that can be used to improve HIV care through medical evaluation to detect and manage DM and dyslipidaemia.
- Our sampling method, which is likely to restrict representativeness, is presumed to make selection bias inevitable. In addition, as a cross-sectional design is used, the study shows no temporal relationships, so the observed associations might not necessarily be causal.

## Introduction

### Background

The HIV pandemic has continued to spread worldwide.^1^ The burden of disease is high in sub-Saharan Africa, where 22.9 million adults and children are affected.^2^ In Ethiopia, an estimated 222 723 people were receiving antiretroviral treatment (ART) in 2010.^2^ Although ART resulted in a remarkable overall improvement in life expectancy and a decreasing trend in mortality, the long-term negative effects of HIV in the era of ART have continued to be a huge challenge. Opportunistic infections and treatment-related complications are still the major causes of morbidity and mortality in HIV-infected individuals in low-income countries, including Ethiopia. Non-communicable diseases (NCDs), such as diabetes mellitus (DM), cardiovascular disease (CVD) and cerebrovascular disease are also being encountered more frequently in the HIV-infected population.^3^

DM is generally emerging as the major non-infectious comorbid condition in HIV-infected individuals. This phenomenon may threaten to reverse the success achieved so far in the care of HIV patients by imposing additional DM-related morbidities and mortalities which are known to complicate not only the medical management but also the economic and policy aspects of HIV care.^2^ In addition to the direct effect of HIV, ART and opportunistic diseases are assumed to contribute to the rise in the occurrence of DM in HIV-infected individuals.^4^ Diabetes is already being reported as a major comorbidity in HIV-infected individuals, and an increasing trend of DM occurrence is being observed in areas of the world where HIV prevalence is high.^5^ ^6^

Since HIV-infected individuals are living longer with ART and improved HIV care, a rise in NCDs in this population is inevitable. There are growing concerns about complications related to the longer use of ART.^7–9^ Furthermore, most people with a metabolic syndrome have insulin resistance, which confers an increased risk on type 2 diabetics. When diabetes becomes clinically apparent, the CVD risk rises sharply.^10^

Even though DM and other metabolic complications are impending challenges in countries with high HIV prevalence, very little evidence exists on the disease burden of DM in the HIV-infected population in the study area.

### Objective

The objective of this study was to assess the prevalence of DM and associated factors among HIV-infected adults in northwest Ethiopia.

## Methods

### Study design

A hospital-based quantitative cross-sectional method was used. All adult HIV-infected individuals who visited the HIV clinic of the University of Gondar Hospital for follow-up care from December 2013 to February 2014 were studied.

### Setting

The study was conducted at the University of Gondar Hospital, which is the only referral hospital in Gondar City, the Amhara National Regional State, Ethiopia. More than 450 000 people visit the hospital every year for different health services. The hospital also serves as a referral centre for a population of five million in the surrounding catchment area which has varying climatic and geographical distributions.

### Participants

Patients over 15 years of age who attended the centre for follow-up care for more than 12 months were included. Pregnant women, patients with any acute illness that required medical or surgical treatment or admission, patients with a clinical thyroid disease, and subjects with chronic hepatitis were excluded from the study. The participants were selected using a systematic sampling procedure; every second person who visited the clinic was selected for the study on a daily basis.

### Variables

A DM diagnosis was based on fasting plasma glucose ≥126 mg/dL, confirmed by repeating the test on another day, or known DM for which treatment was being received. The pre-ART group were subjects who were naïve for ART or who experienced ART either during pregnancy or during post-exposure prophylaxis. These were study subjects who had not received a therapeutic dose of ART or had not received ART for more than 2 months. The ART group were those who had received standard ART regimens according to the national guidelines for more than 8 weeks (2 months). Reduced high-density lipoprotein (HDL) cholesterol (<40 mg/dL in men and <50 mg/dL in women), high low-density lipoprotein (LDL) cholesterol (≥130 mg/dL), clinical information about CD4 counts, drug regimens, medical history, disease status and outcomes were gathered from the follow-up medical records of the ART clinic and from a biochemical (fasting blood sugar, triglyceride and total cholesterol) test. Venous blood (10 mL) was collected from each subject after an overnight fast (at least 8 hours) using standard laboratory techniques.^10^

### Data sources/measurement

Data were gathered using a pretested structured WHO STEPwise instrument approach.^11^ Sociodemographic and socioeconomic data were collected using a structured questionnaire. Fasting blood samples were collected from each patient, and the samples taken during data collection were sent to laboratories for chemical analysis. Six laboratory technicians, four nurses and two supervisors were trained by the principal investigator. Biochemical tests were carried out using a 902 Automatic Analyzer with the Roche/Hitachi kit.

### Sample size

The selected final sample size was calculated on the basis of the following assumptions: use of the single-proportion formula with a 95% confidence level; 2% margin of error; and an expected DM prevalence of 6.4%, the latter based on a study conducted in southwest Ethiopia.^5^ The final sample size was 483, as calculated using Epi Info V.7. Every person aged 15 years and above was selected from 40–60 HIV and AIDS patients who visited the clinic every day. Systematic random sampling was used to collect data. Every second patient who came to the ART clinic was interviewed. The WHO classification guidelines for laboratory tests of fasting blood sugar, lipid profile, lifestyle and physical measurements were used. Standardised techniques and calibrated equipment were used to take physical measurements, such as weight and height; the measurements were taken twice, and the average was used in the analysis.

### Quantitative variables

Body mass index (BMI) was calculated as the ratio of weight in kilograms to the square of height in metres. Waist girth was measured (to the nearest 0.5 cm) by placing a plastic tape horizontally midway between the 12th rib and the iliac crest on the mid-axillary line. Hip circumference was measured around the widest portion of the buttocks, with the tape parallel to the floor.^10^ Waist circumference was classified as normal if it was 93.9 cm or less for men and 79.9 cm or less for women, and high if it was 94 cm or more for men and 80 cm or more for women.^12^ Lipid status was determined using the USA National Cholesterol Education Program total cholesterol ≥200 mg/dL, HDL-cholesterol <40 mg/dL, raised LDL-cholesterol (≥130 mg/dL) and triglycerides ≥150 mg/dL.^13^

### Statistical methods

Data were reported as mean±SD for continuous variables, and as percentages for categorical variables. Proportion estimations of men and women were made. For significance testing, continuous variables were compared using an independent t-test, and categorical data were compared using a χ^2^ test. Logistic regression analysis was applied to determine the associations of established risk factors for DM; ORs with a 95% CI for risk indicators were calculated assuming the lowest prevalence of clinically relevant criteria as a reference value.

### Ethics statement

The study was approved by the institutional review board of the University of Gondar. Subjects were recruited after they were given a clear explanation about the objectives of the study. Subjects who volunteered to participate in the study were included after they had signed a written agreement prepared in Amharic, the local language. For participants who were younger than 18 years and older than 15, written consent was obtained from their guardian, while verbal assent or agreement was obtained from the participant. All participants were informed of their rights to withdraw from the study at any stage or to restrict their data in the analysis. The interview took place in a separate room in order to ensure participants’ right to privacy. The participants were informed that the recorded information would be kept confidential and no name was recorded on the questionnaire. All data were kept safely in a locked cabinet after the analysis. Confidentiality was maintained at all levels of the study.

## Results

### Participants

A total of 483 study participants were enrolled initially, but 21 were excluded because they refused to give blood samples. With a response rate of 95.6%, 462 participants were included in the analysis. Two hundred and ninety-five of the participants (63.9%) had been taking ART for at least 12 months, and the remaining 167 (36.1%) were in pre-ART care.

### Descriptive data

The mean±SD age of the participants taking ART was 37.5±9.2 years and that of the pre-ART participants was 34.6±9.9 years. One hundred and forty-two (30.7%) of the participants were male, 83 (58.4%) of whom were taking ART. Two hundred and twelve (66.2%) of the female participants were receiving ART during the study. Two hundred and three (43.9%) of the participants were married, and 135 (66.5%) of these were receiving ART. Three hundred and two (65.4%) participants did not drink alcohol, and 435 (94.2%) smoked no cigarettes of any kind (table 1). The proportion with a large waist circumference was 36.5%. Of the 310 ART cases, the regimen ‘AZT+3TC+NVP’ was the most commonly prescribed treatment at the hospital (157; 50.6%). The mean±SD value for the most recent CD4 count determined within the previous 3 months was 412±195 cells/µL for participants receiving ART, and 369±218 cells/µL for those who were on pre-ART care. Fifty-nine (12.8%) participants were either overweight or obese; 40 (13.6%) of these were receiving ART, and 19 (11.4%) were on pre-ART care (table 1).

**Table 1:** Sociodemographic and clinical characteristics of HIV-infected adults by ART status at the Hospital of Gondar University, northwest Ethiopia (2014)

Variable	ART	Pre-ART	Both
Age (years)			
<25	19 (6.4)	31 (18.6)	50 (10.0)
25–34	101 (34.2)	56 (33.5)	157 (33.9)
35–44	113 (38.3)	53 (31.7)	166 (35.9)
45 and older	62 (21.0)	27 (16.3)	89 (19.3)
Sex			
Men	83 (58.4)	59 (41.6)	142 (30.7)
Women	212 (66.2)	108 (33.8)	320 (69.3)
Religion			
Orthodox	269 (63.4)	155 (36.6)	421 (91.8)
Muslim	26 (70.3)	11 (29.7)	37 (8.0)
Other	0 (0.00)	1 (100)	1 (0.2)
Marital status			
Never married	21 (36.8)	36 (63.2)	57 (12.3)
Currently married	135 (66.5)	68 (33.5)	203 (43.9)
Divorced	51 (68.0)	24 (32.0)	75 (16.2)
Widowed	61 (69.3)	27 (30.7)	88 (19.1)
Separated	27 (69.2)	12 (30.8)	39 (8.4)
Alcohol consumption			
Yes	61 (38.1)	99 (61.9)	160 (34.6)
No	234 (77.5)	68 (22.5)	302 (65.4)
Smoking			
Yes	7 (25.9)	20 (74.1)	27 (5.8)
No	288 (66.2)	147 (33.8)	435 (94.2)
BMI			
Underweight	49 (51.0)	47 (48.9)	96 (20.8)
Normal weight	206 (67.1)	101 (32.9)	307 (66.4)
Overweight	31 (67.4)	15 (32.6)	46 (9.9)
Obese	9 (69.2)	4 (30.8)	13 (2.8)

Values are n (%).

ART, antiretroviral treatment; BMI, body mass index.

### Outcome data

**Figure 1:**
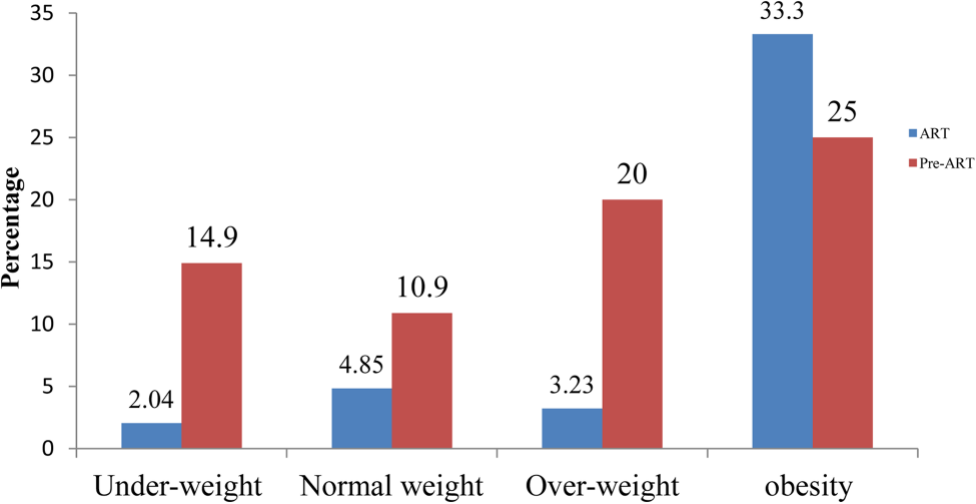
Proportions of diabetes mellitus in relation to body mass index among HIV-infected adults receiving antiretroviral treatment (ART) and on pre-ART care at the Hospital of Gondar University, northwest Ethiopia (2014).

A total of 37 (8%; 95% CI 5.5% to 10.5%) participants were found to have DM; 22 (13.2%; 95% CI 8.0% to 18.3%) of these were on pre-ART care, while 15 (5.1%; 95% CI 2.6% to 7.6%) were receiving ART. All DM cases diagnosed during the study were type 2. The mean±SD age of subjects with DM was 38.4±9.9 years. Thirteen (35.1%) of the 37 diabetics who were previously undiagnosed were newly diagnosed with DM. Two-hundred and two (43.7%) were widowed, separated or divorced. Fifteen (10.6%) of the male participants and 22 (6.9%) of the female participants were diabetic (table 2). Forty (8.7%; 95% CI 6.1% to 11.2%) participants had been previously treated for tuberculosis (TB), and five (12.5%) of these were found to be diabetic. Seventy-nine (17.1%) participants were found to be hypertensive, and 16 (20.3%) of these were also diabetic. Of the 37 diabetic patients, 16 (43.2%) were also hypertensive, whereas only 63 (14.8%) of the non-diabetic participants were hypertensive (table 2). The occurrence of DM has shown an increasing pattern among obese participants in general, with an even higher degree among those receiving ART (figure 1).

**Table 2:** Multivariate analysis of factors associated with diabetes mellitus among HIV-infected adults at the Hospital of Gondar University, northwest Ethiopia (2014)

	Diabetes mellitus		Crude OR (95% CI)	Adjusted OR (95% CI)
Variable	Yes, n (%)	No, n (%)		
Sex				
Male	15 (10.6)	127 (89.4)	1.00	1.00
Female	22 (6.9)	298 (93.1)	0.62 (0.31 to 1.24)	1.11 (0.45 to 2.70)
Education				
Illiterate†	7 (5.3)	124 (94.7	1.00	1.00
Grade 1–6	3 (3.5)	82 (94.5)	0.65 (0.16 to 2.58)	0.47 (0.11 to 2.06)
Grade 7–12	20 (10.8)	166 (89.2)	2.13 (0.87 to 5.21)	1.88 (0.71 to 4.94)
Diploma and above	5 (27.8)	13 (72.2)	6.81 (1.89 to 24.55)*	11.8 (2.28 to 61.4)*
History of TB prescription				
No	32 (7.6)	390 (92.4)	1.00	1.00
Yes	5 (12.5)	35 (87.5)	1.74 (0.64 to 4.75)	1.52 (0.47 to 4.96)
ART status				
ART	15 (5.08)	280 (94.9)	1.00	1.00
Pre-ART	22 (13.2)	145 (86.8)	2.83 (1.42 to 5.62)*	4.47 (1.80 to 11.08)*
BMI (kg/m^2^)				
Underweight	8 (8.3)	88 (91.7)	1.00	1.00
Normal weight	21 (6.84)	286 (93.2)	0.81 (0.34 to 1.89)	0.95 (0.35 to 2.58)
Overweight	4 (8.7)	42 (91.3)	1.05 (0.29 to 3 to 67)	0.75 (0.17 to 3.24)
Obese	4 (30.8)	9 (69.2)	4.89 (1.23 to 19.48)*	6.55 (1.20 to 35.8)*
Hypertension				
No	21 (5.5)	362 (94.5)	1.00	1.00
Yes	16 (20.3)	63 (79.7)	4.38 (2.16 to 8.84)*	3.45 (1.50 to 7.90)*
HDL-cholesterol				
Low	24 (8.00)	276 (92.0)	1.00	1.00
High	13 (8.02)	149 (91.9)	0.99 (0.49 to 2.01)	1.10 (0.48 to 2.52)
LDL-cholesterol				
Normal	30 (7.9)	350 (92.1)	1.00	1.00
High	7 (8.54)	75 (91.6)	1.09 (0.46 to 2.57)	0.91 (0.32 to 2.55)
Triglyceride				
Normal	20 (6.08)	309 (93.9)	1.00	1.00
High	17 (12.8)	116 (87.2)	2.26 (1.14 to 4.47)*	2.24 (1.02 to 49.5)*

*p<0.05.

ART, antiretroviral treatment; BMI, body mass index; HDL, high-density lipoprotein; LDL, low-density lipoprotein; TB, tuberculosis.

Low HDL values (HDL-cholesterol <40 mg/dL) were observed in 64.9% (95% CI 60.6% to 69.3%) of the study population. The percentage with high triglyceride (≥150 mg/dL) was 28.8%; high LDL-cholesterol (≥130 mg/dL) was observed in 17.7%, and patients receiving ART had a higher proportion (23.7%) of LDL-cholesterol than those on pre-ART care (7.2%). The proportion with total cholesterol >200 mL/dL was 18.6%. This was slightly higher among patients receiving ART (23.1%; 95% CI 15.9% to 30.1%) than among those on pre-ART care (10.6%; 95% CI 4.1% to 17.3%). While the percentage with hypertriglyceridaemia was slightly higher for pre-ART patients (30.5%) than for those receiving ART (27.8%), 90.2% of the ART and 95.2% of the pre-ART patients had at least one laboratory abnormality for the diagnosis of dyslipidaemia.

In the multivariate logistic regression analysis, the odds of having DM were more than six times higher among obese individuals than among the underweight (adjusted OR (AOR) 6.55; 95% CI 1.20 to 35.8). The odds of being diabetic were three times higher among hypertensive HIV patients than among normotensive HIV patients (AOR 3.45; 95% CI 1.50 to 7.90). The occurrence of DM was four times higher in individuals who were not receiving ART than in those who were receiving ART (AOR 4.47; 95% CI 1.80 to 11.08). The occurrence of DM in individuals with hypertriglyceridaemia was twice as high as in the group of patients with normal triglyceride concentration (AOR 2.24; 95% CI 1.02 to 49.5). Diploma and degree level education was also significantly associated with DM (AOR 11.8; 95% CI 2.28 to 61.4) (table 2).

## Discussion

### Key result

This study has identified a high prevalence of DM among HIV patients, and the proportion of DM is higher among pre-ART and hypertensive patients. Obesity, hypertension, pre-ART status, hypertriglyceridaemia and higher educational level are the strongest predictors of DM in HIV and AIDS patients. The prevalence of diabetes is higher in both pre-ART (13.2%) and ART (5.1%) cases, which is similar to a finding from Spain.^14^ The prevalence of DM in the ART group is slightly lower than in a report from Jimma in the southwestern part of the country (8.5%), while the prevalence among pre-ART groups is higher than in the report from the southwest (0.9%).^5^ The observed high prevalence of DM in the pre-ART group is confounded by a family history of DM; in this study, the number with a family history of DM in the pre-ART group was 13 (8.18%), while it was 11 (4.01%) in the ART group. This implies that a family history of DM contributed to the high prevalence in the pre-ART group. Another study has shown that the prevalence of type 2 DM is five- to nine-fold greater in HIV-infected individuals than in HIV-negative individuals.^8^ This might be partly explained by previous reports showing higher occurrence of insulin resistance in HIV patients, which was assumed to be a consequence of abnormal fat metabolism and distribution in such patients.^15^ ^16^ Similarly, various studies have shown a rise in abdominal obesity to be associated with DM and hypertriglyceridaemia,^17^ ^18^ which is in line with our finding of a markedly high proportion of DM in the obese pre-ART group. This probably explains the observed high prevalence of DM in subjects with high BMI. Thirteen (35.1%) of the DM cases were newly identified during the study. This may be explained by the fact that type 2 DM can be asymptomatic for a long time until even chronic complications have occurred. Another plausible explanation for the high proportion (35%) of DM cases newly diagnosed in the study is the fact that DM screening is not part of the routine care of HIV patients in this setting.

There is growing concern about the long-term complications of HIV treatment related to NCD comorbidities, including DM, dyslipidaemia, cerebrovascular disease and CVD.^9^ ^19^ The current high prevalence of HIV patients in low- and middle-income countries may imply that more individuals are vulnerable to diabetes, and this will directly negatively affect the success and progress of clinical and public health interventions in the HIV-infected population. Therefore, the management of HIV-infected patients requires careful consideration of the comorbidity of DM to ensure beneficial and safe treatment.

The majority of HIV and AIDS patients have had at least one lipid profile abnormality that can label them as having dyslipidaemia, and this is in line with another study conducted in southern Ethiopia.^5^ Similarly, another study shows that the natural course of HIV infection is characterised by reductions in HDL-cholesterol.^5^ Other studies also indicate that weight loss and protein depletion contribute to the reduction in HDL-cholesterol in this group of patients.^20^ ^21^ Although not statistically significant, diabetes was more common (12.5%) among subjects with a history of treatment for TB than in those without such a history (7.6%). This may be due to the impact of TB on DM prevalence, as DM may increase the probability of contracting TB.^22^ ^23^

### Limitations

Owing to the cross-sectional design of the study, we are not able to determine temporal relationships between DM and the associated factors. Longitudinal research is needed to determine temporal and causal relationships between the covariates and DM in HIV-infected individuals. Also the sample size was not sufficient to produce enough diabetic cases to carry out detailed analysis of the various risk factors. The observed high prevalence of DM among HIV-positive patients with higher educational attainment may be confounding. As increasing age has a linear relationship with level of education, an individual with greater age may have more chance of developing DM than a younger one. This study can be regarded as an eye-opener for studies on DM in the HIV-positive population of Ethiopia.

## Conclusion

The prevalence of DM and dyslipidaemia is high among HIV-infected individuals, particularly those in the pre-ART group. A significant proportion (35%) of DM cases was newly diagnosed during the study. Finally, among HIV-infected adults, higher education, hypertension and obesity were found to be associated with DM. We recommend that due attention be given to the impact of DM during clinical and health policy decisions in order to effectively maintain the success and progress achieved so far in the fight against HIV. We suggest that DM screening be part of the routine care at HIV clinics across the country.

## References

[1] WHO. WHO report on global surveillance of epidemic-prone infectious diseases. 2000.

[2] WHO. GLOBAL HIV/AIDS RESPONSE: Epidemic update and health sector progress towards Universal Access, 2011.

[3] Kalra S, Agrawal N Diabetes and HIV: current understanding and future perspectives. Curr Diab Rep 2013;13:419–27. 10.1007/s11892-013-0369-9 23446780

[4] Paula AA, Schechter M, Tuboi SH Continuous increase of cardiovascular diseases, diabetes, and non-HIV related cancers as causes of death in HIV-infected individuals in Brazil: an analysis of nationwide data. PLoS ONE 2014;9:e94636 10.1371/journal.pone.0094636 24728320 PMC3984254

[5] Mohammed AE, Shenkute TY, Gebisa WC Diabetes mellitus and risk factors in human immunodeficiency virus-infected individuals at Jimma University Specialized Hospital, Southwest Ethiopia. Diabetes Metab Syndr Obes 2015;8:197–206. 10.2147/DMSO.S80084 25926749 PMC4403746

[6] Tripathi A, Liese AD, Jerrell JM Incidence of diabetes mellitus in a population-based cohort of HIV-infected and non-HIV-infected persons: the impact of clinical and therapeutic factors over time. Diabet Med 2014;31:1185–93. 10.1111/dme.12455 24673640

[7] Vugt MV, Hamers R, Schellekens O Diabetes and HIV/AIDS in sub-Saharan Africa: the need for sustainable healthcare systems. Diabetes Care 2007;52(3):23–6.

[8] Samaras K, Wand H, Law M Prevalence of metabolic syndrome in HIV-infected patients receiving highly active antiretroviral therapy using International Diabetes Foundation and Adult Treatment Panel III criteria: associations with insulin resistance, disturbed body fat compartmentalization, elevated C-reactive protein, and [corrected] hypoadiponectinemia. Diabetes Care 2007;30:113–19. 10.2337/dc06-1075 17192343

[9] Behrens GMN, Meyer-Olson D, Stoll M Clinical impact of HIV-related lipodystrophy and metabolic abnormalities on cardiovascular disease. AIDS 2003;17(Suppl 1):S149–S54. 10.1097/00002030-200304001-00018 12870541

[10] Grundy SM, Brewer HB Jr, Cleeman JI Definition of metabolic syndrome: Report of the National Heart, Lung, and Blood Institute/American Heart Association Conference on Scientific Issues Related to Definition. Circulation 2004;109:433–8. 10.1161/01.CIR.0000111245.75752.C6 14744958

[11] Longo-Mbenza B, Ngoma DV, Nahimana D Screen detection and the WHO stepwise approach to the prevalence and risk factors of arterial hypertension in Kinshasa. Eur J Cardiovasc Prev Rehabil 2008;15:503–8. 10.1097/HJR.0b013e3282f21640 18830083

[12] Lau DC, Douketis JD, Morrison KM 2006 Canadian clinical practice guidelines on the management and prevention of obesity in adults and children [summary]. CMAJ 2007;176:S1–13. 10.1503/cmaj.061409 PMC1839777 17420481

[13] National Cholesterol Education Program (NCEP) Expert Panel on Detection, Evaluation, and Treatment of High Blood Cholesterol in Adults (Adult Treatment Panel III). Third Report of the National Cholesterol Education Program (NCEP) Expert Panel on Detection, Evaluation, and Treatment of High Blood Cholesterol in Adults (Adult Treatment Panel III) final report. Circulation 2002;106:3143–421. 12485966

[14] Araujo S, Bañón S, Machuca I Prevalence of insulin resistance and risk of diabetes mellitus in HIV-infected patients receiving current antiretroviral drugs. Eur J Endocrinol 2014;171:545–54. 10.1530/EJE-14-0337 25117462

[15] Behrens G, Dejam A, Schmidt H Impaired glucose tolerance, beta cell function and lipid metabolism in HIV patients under treatment with protease inhibitors. AIDS 1999;13:F63–70. 10.1097/00002030-199907090-00001 10416516

[16] Carr A, Samaras K, Thorisdottir A Diagnosis, prediction, and natural course of HIV-1 protease-inhibitor-associated lipodystrophy, hyperlipidaemia, and diabetes mellitus: a cohort study. The Lancet 1999;353:2093–9. 10.1016/S0140-6736(98)08468-2 10382692

[17] Després JP, Lemieux I Abdominal obesity and metabolic syndrome. Nature 2006;444:881–7. 17167477 10.1038/nature05488

[18] Grinspoon S Mechanisms and strategies for insulin resistance in acquired immune deficiency syndrome. Clin Infect Dis 2003;37:S85–90. 10.1086/375885 12942379

[19] Kissebah AH, Krakower GR Regional adiposity and morbidity. Physiol Rev 1994;74:761–811. 7938225 10.1152/physrev.1994.74.4.761

[20] Grunfeld C, Pang M, Doerrler W Lipids, lipoproteins, triglyceride clearance, and cytokines in human immunodeficiency virus infection and the acquired immunodeficiency syndrome. J Clin Endocrinol Metab 1992;74:1045–52. 10.1210/jcem.74.5.1373735 1373735

[21] Haugaard SB, Andersen O, Pedersen SB Tumor necrosis factor α is associated with insulin-mediated suppression of free fatty acids and net lipid oxidation in HIV infected patients with lipodystrophy. Metab Clin Exp 2006;55:175–82. 10.1016/j.metabol.2005.08.018 16423623

[22] Young F, Critchley JA, Johnstone LK A review of co-morbidity between infectious and chronic disease in Sub Saharan Africa: TB and diabetes mellitus, HIV and metabolic syndrome, and the impact of globalization. Global Health 2009;5:9 10.1186/1744-8603-5-9 19751503 PMC2753337

[23] Singla R, Khan N, Al-Sharif N Influence of diabetes on manifestations and treatment outcome of pulmonary TB patients. Int J Tuberc Lung Dis 2006;10:74–9. 16466041

